# Identifying metastasis-initiating miRNA-target regulations of colorectal cancer from expressional changes in primary tumors

**DOI:** 10.1038/s41598-020-71868-0

**Published:** 2020-09-10

**Authors:** Jongmin Lee, Hye Kyung Hong, Sheng-Bin Peng, Tae Won Kim, Woo Yong Lee, Seong Hyun Yun, Hee Cheol Kim, Jiangang Liu, Philip J. Ebert, Amit Aggarwal, Sungwon Jung, Yong Beom Cho

**Affiliations:** 1 Gachon Institute of Genome Medicine and Science, Gachon University Gil Medical Center, Incheon, Republic of Korea; 2 Research Institute for Future Medicine, Samsung Medical Center, Seoul, Republic of Korea; 3 Eli Lilly and Company, Indianapolis, IN USA; 4 Department of Health Sciences and Technology, SAIHST, Sungkyunkwan University, Seoul, Republic of Korea; 5 Department of Surgery, Samsung Medical Center, Sungkyunkwan University School of Medicine, Seoul, Republic of Korea; 6 Department of Genome Medicine and Science, Gachon University College of Medicine, Incheon, Republic of Korea

**Keywords:** Cancer genomics, Computational biology and bioinformatics, Colorectal cancer, Metastasis

## Abstract

Colorectal cancer (CRC) is prevalent with high mortality, with liver metastasis contributing as a major factor that worsens the survival of patients. The roles of miRNAs in CRC have been elucidated, subsequent to recent studies that suggest the involvement of miRNAs in cancer biology. In this study, we compare the miRNA and gene expression profiles of primary tumors between two groups of patients (with and without liver metastasis) to identify the metastasis-initiating microRNA-target gene regulations. Analysis from 33 patients with metastasis and 14 patients without metastasis revealed that 17 miRNAs and their 198 predicted target genes are differentially expressed, where the target genes showed association with cancer progression and metastasis with statistical significance. In order to evaluate the clinical implications of the findings, we classified CRC patients of independent data into two groups based on the identified miRNA-target regulations, where one group was closer to primary tumors with metastasis than the other group. The comparison of survival showed statistically significant difference, thereby implying the roles of the identified miRNA-target regulations in cancer progression and metastasis. The identification of metastasis-initiating miRNA-target regulations in this study will lead to better understanding of the roles of miRNAs in CRC progression.

## Introduction

CRC is one of the most common cancers worldwide. According to a study in 2018^1^, the number of CRC incidences worldwide ranked third, with mortality being second highest among all cancers. Numerous studies have endeavored to unravel the clinical characteristics and the mechanisms of progression for CRC, and liver metastasis was identified as one of the major prognostic factors affecting the survival rate of patients^2^. Studies have investigated liver metastasis of CRC based on genomic profiles, where gene expressions and DNA somatic mutations were commonly used together with clinical characteristics of the patient^3–8^. In addition to expressions and mutations of genes, the role of miRNAs in CRC has been also elucidated^9^. miRNAs are short non-coding RNAs composed of 21–25 nucleotides. At least 60% of the human genes are known to be regulated by miRNAs^10^, and recent studies suggest that miRNAs are also involved in various cancer biologies^11–14^.

Previous studies investigated the role of miRNAs associated with metastasis in CRC. Lin et al. identified metastasis-related miRNAs from primary colorectal cancer based on the miRNA expression profiles from microarrays^5^. Goossens-Beumer et al. analyzed the expression levels of miRNAs from primary CRC tissues with small RNA-sequencing to classify miRNAs that predict metastasis of colon cancers^6^. Pizzini et al. obtained miRNA and mRNA expression data with microarray profiling, and compared their profiles in primary and metastatic tumors, and normal colon tissues^7^. Röhr et al. analyzed high-throughput sequencing data of miRNAs and mRNA transcripts from normal colon and metastasis tumor samples^8^, and they evaluated the up/down-regulations of miRNAs from individual patients to identify candidates of therapeutic targets. Although these studies identified miRNAs or their target regulations associated with metastasis, they have certain limitations. Lin et al.^5^ and Beumer et al.^6^ investigated the expression of miRNAs but did not consider gene expressions that can be potential targets of miRNAs. Pizzini et al.^7^ analyzed the expressions of miRNAs and genes together, but their study has limited miRNA coverage as they used microarrays that do not fully represent the currently known miRNAs. Since Beumer et al.^6^ used miRNA expression profiles from small RNA-seq, their study covered most of the known miRNAs, but they did not consider mRNA expression profiles from matched samples. Another study^8^ compared miRNA and mRNA expression profiles from primary colon tumor tissues and liver metastatic tumor tissues, but was more focused on identifying differentiated miRNA-gene regulations between primary and metastatic tumors rather than identifying miRNA-gene regulations that initiate the liver metastasis of CRC.

In this study, we use miRNA expression data obtained from small RNA-seq, together with whole transcriptome sequencing (WTS)-based gene expression profiles from matched tumor tissues. By investigating miRNA and mRNA profiles from primary tumor tissues with and without accompanying liver metastasis, we aim to identify miRNAs and their potential target genes that show altered expressions and may contribute to the initiation of liver metastasis. Our study will provide more convincing knowledge about the roles of miRNA-gene regulations that initiate liver metastasis in CRC.

## Methods

### Sample preparation and expression profiling

Primary tumor tissues were obtained from 47 CRC patients and prepared for miRNA and mRNA expression profiling. Of the 47 patients, 14 showed no metastasis, while 33 patients had accompanying liver metastasis. The detailed demographic and clinical characteristics of the 47 patients are given in Table 1. The two groups of patients did not show statistically significant differences in ages, sex, tumor locations, and the degree of cancer cell differentiation (See Supplementary Table S1 online for detailed information). All samples were surgically acquired before any treatment. Both of small RNA-seq and whole transcriptome-sequencing were conducted for these 47 primary tumor samples on Illumina HiSeq 2000. Whole transcriptome-sequencing was done using the Illumina TruSeq RNA Sample Preparation Kit v2, and paired-end sequencing with a read length of 100 bp and targeted read depth of 50 million reads/sample was performed. On average, the Q30 sequence quality was 93.26% and 87.17% of data was mapped to genome. Small RNA-seq was conducted as single-end sequencing with a read-length of 50 bp and targeted read depth of 10 million reads/sample. On average, the Q30 sequence quality was 94.66% and 86.71% of data was mapped to genome. Sequences were aligned to the human reference genome (hg19) with the Burrows-Wheeler Aligner (https://bio-bwa.sourceforge.net/). Expression values were normalized using RNA-Seq by Expectation–Maximization (RSEM) (https://deweylab.github.io/RSEM/). From sequencing, expression levels of 2,669 miRNAs and 23,956 genes were acquired. While the previous studies^5^ that evaluated miRNA expressions with microarrays had limited miRNA coverage of only up to 1,146 miRNAs, we profiled the expression levels of 2,669 miRNAs that covers majority of the known 2,883 human miRNAs^15^. The experiments conducted on patient samples were approved by the institutional review board of Samsung Medical Center (2010-04-004). Written informed consents were obtained from all participating patients. All experiments and analysis procedures were performed in accordance with the relevant guidelines and regulations.

**Table 1:** Demographic and clinical landscape of 47 CRC patients.

		Without metastasis	With liver metastasis
Number		14	33
Age	≤ 60	10 (71.4%)	21 (63.6%)
	> 60	4 (28.6%)	12 (36.4%)
Sex	Male	7 (50.0%)	16 (48.5%)
	Female	7 (50.0%)	17 (51.5%)
Tumor location	Colon	13 (92.9%)	24 (72.7%)
	Rectum	1 (7.1%)	9 (27.3%)
Cell differentiation	Adenocarcinoma		
	W/D	1 (7.1%)	3 (9.1%)
	M/D	11 (78.7%)	27 (81.8%)
	P/D	1 (7.1%)	2 (6.1%)
	Mucinous carcinoma	1 (7.1%)	1 (3.0%)
Stage	II	7 (50.0%)	0 (0%)
	III	7 (50.0%)	0 (0%)
	IV	0 (0%)	33 (100%)
Invasion	Lymphatic	5 (35.7%)	18 (54.5%)
	Perineural	3 (21.4%)	20 (60.6%)
	Vascular	3 (21.4%)	16 (48.5%)
MSI status	MSS	11 (78.6%)	32 (97.0%)
	MSI	3 (21.4%)	0 (0%)
	Undesecribed	0 (0%)	1 (3.0%)

W/D, well differentiated; M/D, moderately differentiated; P/D, poorly differentiated; MSI, microsatellite instability; MSS, microsatellite stable.

### Identifying metastasis-initiating miRNA-target regulations

In order to identify miRNAs and genes that show differential expression between patients with and without liver metastasis, we compared the expressions of miRNAs and genes between primary tissues with liver metastasis and without metastasis. Differential expressions between the two groups were evaluated with raw transcript read counts using the edgeR^16^ R package, one of the most popular software tools for identifying differentially expressed RNAs from sequencing data, and the resultant *p* values of statistical significance were adjusted with the Benjamini–Hochberg method^17^. In this study, a miRNA or a gene is determined to be differentially expressed (DEmiRNA or DEG) if it shows greater than or equal to twofold change in the average expression level between the tumors with and without metastasis, and its adjusted *p* value is less than 0.05. A power analysis suggested more stringent criteria for differential expressions, especially for miRNAs, but we maintained these thresholds as more stringent criteria were too restrictive in our data configuration and considering the technical difficulties in practice of obtaining miRNA sequencing data of similar amount with RNA-seq data (see Supplementary Figs. S1–S3 online).

**Figure 1:**
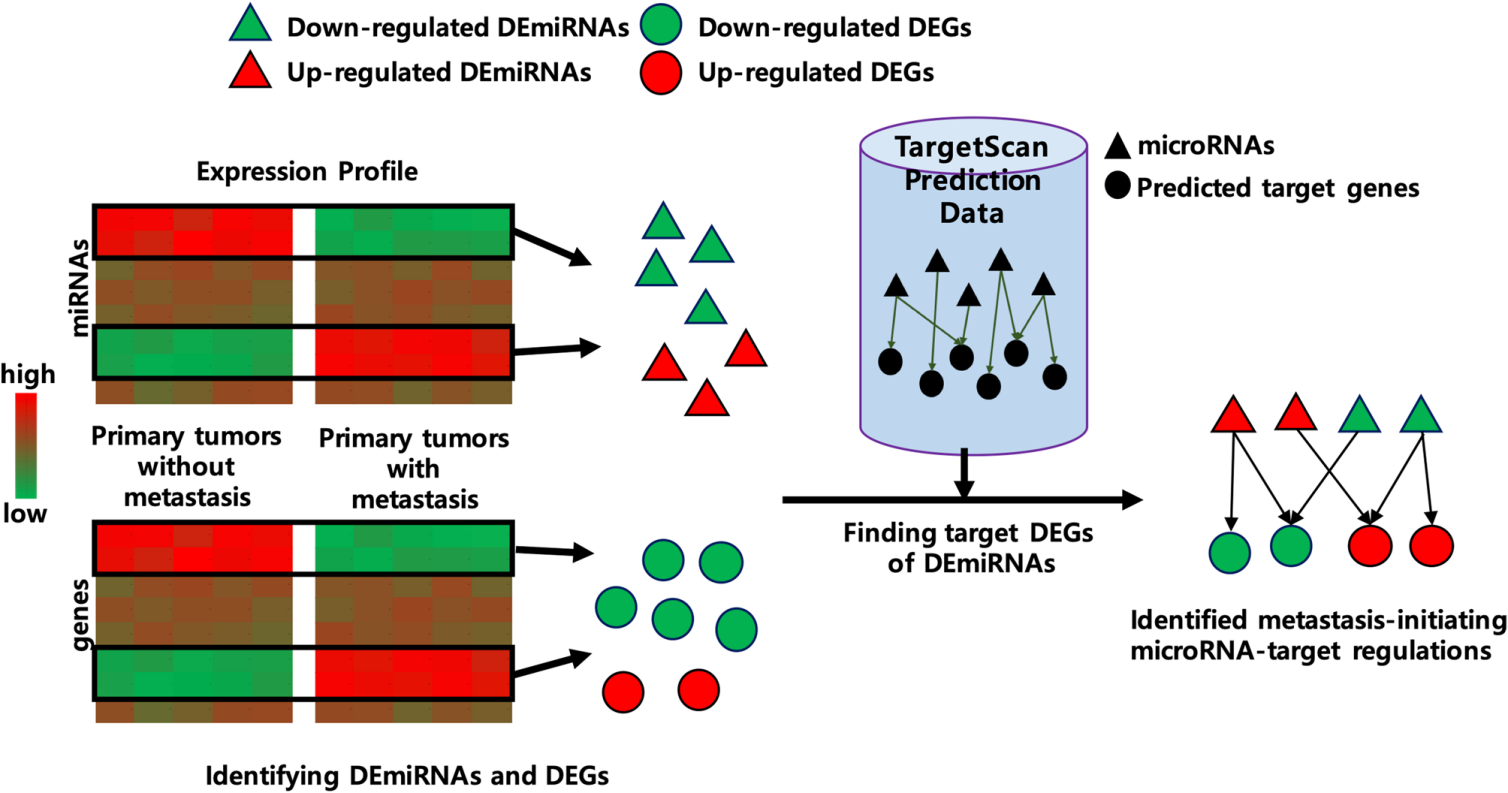
Research overview. Two sample groups of primary tumors, with and without metastasis, are compared, to find metastasis-initiating miRNA-target regulations. DEmiRNAs and DEGs are identified if they show differential expressions between the two groups of samples. The pairs of DEmiRNAs and genes that have predicted miRNA-target relationships are chosen as metastasis-initiating miRNA-target regulations from primary tumors.

Based on the DEmiRNAs and DEGs obtained, we identified differentially expressed miRNA-target regulations as metastasis-initiating, by utilizing predicted miRNA-target gene information. The predicted human miRNA-target information from TargetScan v7.2^18^ is used in this study, which encompasses 120,702 predicted human miRNA family-target regulations. For each DEmiRNA, its predicted target genes are identified among the DEGs, and these predicted regulations between the DEmiRNA and its target DEGs constitute miRNA-target regulations that contribute to the initiation of CRC liver metastasis. The overview of entire processes is illustrated in Fig. 1.

### Evaluating the metastasis-specificity of identified miRNA-target regulations

We evaluated the metastatic association and statistical significance of the identified miRNA-target regulations. In order to evaluate the association with metastasis, we applied the functional enrichment test to the identified target genes to evaluate their role in biological functions related to metastasis. Using the MSigDB^19^, 22 hallmark gene sets (Supplementary Table S2 online) were selected as representative factors related to metastatic biological functions. Hypergeometric *p* values were evaluated for each gene set with the identified target genes from the identified metastasis-initiating regulations, and adjusted with the Benjamini–Hochberg method^17^. Adjusted *p* value less than 0.05 for a gene set is indicative of statistically significant enrichment of the function corresponding to the gene set in the target genes. The association of identified target genes to metastasis can be represented by the number of enriched metastasis-related functions from the 22 gene sets: higher number of associated metastasis-related functions in target genes is indicative of greater association with metastasis. We hypothesized that if the identified miRNA-target regulations represent true biology related to metastasis, the target genes should be significantly enriched with metastasis-related functions than by random chance, by showing higher metastasis-relatedness. To verify this hypothesis, we applied a random permutation approach to test if the evaluated metastasis-relatedness of the identified miRNA-target regulations is greater than that obtained by random chance. For each random permutation, the metastasis status of expression samples was randomly shuffled and re-assigned, and the entire process (from identifying metastasis-related miRNA-target regulations to evaluating the metastasis-relatedness) was conducted for the randomly permuted data. By repeating these random permutations for a fixed number of times (1,000 in this study) and examining the cases showing greater than or equal to the original metastasis-relatedness, we estimated the *p* value of the original metastasis-relatedness. More detailed descriptions of this permutation test are given in Supplementary Fig. S4 online with a schematic diagram.

### Evaluating the clinical significance of identified miRNA-target regulations with independent data

The clinical significance of the identified miRNA-target regulations is evaluated using an independent data set, by obtaining miRNA and gene expression profiles of 428 primary tumor samples of CRC from The Cancer Genome Atlas (TCGA)^20^. Since the identified miRNA-target regulations are expected to be related with metastasis initiation, we assume that prognosis of patients will differ, depending on how their miRNA and gene expression profiles resemble the pattern of identified miRNA-target regulations. The expression profiles of each TCGA sample were evaluated with their respective expression levels of miRNAs and genes of the identified regulations, to predict the resemblance of the sample with primary tumors with or without metastasis. Expression values from our own samples are transcripts per million (TPM)-normalized, while gene expression values are fragments per kilobase of exon model per million reads mapped (FPKM)-normalized and miRNA expression values are reads per million reads (RPM)-normalized from TCGA. In order to transform the expression values to be comparable between two data sets, all expression values other than zero expression were z-transformed for each sample and zero expression values were replaced with the smallest z-transformed expression value within each data set. The transformed expression value represents the relative expression of a miRNA/gene within each sample. The expression level of each miRNA or gene of a TCGA sample was then categorized as per its similarity to the expression values of the same miRNA or gene in primary tumors with or without accompanying metastasis from our data set. More specifically, for each miRNA or gene from the identified metastasis-initiating miRNA-target regulations, the representative range *R*_*woM*_ of z-transformed expression was defined from our data set for the sample group *woM* of primary tumors without metastasis, as follows (and vice versa for the sample group *wM* of primary tumors with metastasis):

$$ \begin{aligned}    & R_{{woM}}  \\     & \quad  = \left\{ {\begin{array}{*{20}l}    {\left[ {\mu _{{woM}}  - \sigma _{{woM}} ,~\mu _{{woM}}  + \sigma _{{woM}} } \right],~} \hfill & \begin{gathered}   \quad {\text{if}}\;\mu _{{woM}}  < \mu _{{wM}} \;{\text{and}}\;\mu _{{woM}}  + \sigma _{{woM}}  < \mu _{{wM}}  - \sigma _{{woM}} \;{\text{or}} \hfill \\   \quad \quad \mu _{{woM}}  > \mu _{{wM}} \;{\text{and}}\;\mu _{{woM}}  - \sigma _{{woM}}  > \mu _{{wM}}  + \sigma _{{woM}}  \hfill \\  \end{gathered}  \hfill & {\quad (1)} \hfill  \\    {\left[ {\mu _{{woM}}  - \sigma _{{woM}} ,~\frac{{\left( {\mu _{{woM}}  + \sigma _{{woM}} } \right) + \left( {\mu _{{wM}}  - \sigma _{{wM}} } \right)}}{2}} \right],} \hfill & {\quad {\text{if~}}\;\mu _{{woM}} \left\langle {\mu _{{wM}} \;{\text{and}}\;\mu _{{woM}}  + \sigma _{{woM}} } \right\rangle \mu _{{wM}}  - \sigma _{{woM}} } \hfill & {\quad (2)} \hfill  \\    {\left[ {\frac{{\left( {\mu _{{wM}}  + \sigma _{{wM}} } \right) + \left( {\mu _{{woM}}  - \sigma _{{woM}} } \right)}}{2},~\mu _{{woM}}  + \sigma _{{woM}} } \right],} \hfill & {\quad {\text{if~}}\;\mu _{{woM}}  > \mu _{{woM}} \;{\text{and}}\;\mu _{{woM}}  - \sigma _{{woM}}  > \mu _{{wM}}  + \sigma _{{woM}} } \hfill & {\quad (3)} \hfill  \\   \end{array} } \right., \\  \end{aligned}   $$

where $\mu$ is the average, and $\sigma$ is the standard deviation of z-transformed expressions from the sample group. In these equations, Eq. (1) is applied when there is no overlap between the two representative ranges *R*_*woM*_ and *R*_*M*_, and qs. (2) and (3) are applied when there is overlap between these ranges (see Supplementary Fig. S5 online for further descriptions with schematic illustrations). For each sample from the independent TCGA data, each miRNA or gene from identified regulations is categorized as having similar expression to that of a sample group from our data set, if its z-transformed expression level from the TCGA sample falls within the representative range from our data set for the same miRNA or gene. By repeating this process for all miRNAs and genes of a TCGA case, the number of miRNAs and genes that resemble the expression ranges of primary tumors with metastasis and without metastasis can be counted. A TCGA case is predicted to resemble the primary tumors with metastasis of our data set if it has significantly more miRNA-target genes that have expressions similar to primary tumors with metastasis than miRNAs-target genes that have expressions similar to primary tumors without metastasis (by comparing the number of genes using the chi-square test with a *p* value threshold = 0.05); such TCGA cases constitute a separate group *G*_1_ (vice versa for TCGA cases resembling primary tumors without metastasis, duly categorized as group *G*_2_). A TCGA case is excluded from the analysis if the case has significantly more miRNAs (by the chi-square test with a *p* value threshold = 0.05) that resemble the opposite sample group and contradicting the miRNA-target expression patterns (for example, a case categorized as *G*_1_ by genes but *G*_2_ by miRNAs). After categorizing the independent TCGA cases into two groups representing primary tumors with and without metastasis, the survival periods of patients were compared, to recognize potential relevance with the identified metastasis-initiating miRNA-target regulations. Log-rank test^21^ is used for this survival comparison, and the multivariate Cox proportional hazards (Cox-PH) model is used to adjust hazard ratios for available clinical variables from TCGA.

## Results

### Identification of metastasis initiating miRNA-target regulations

**Figure 2:**
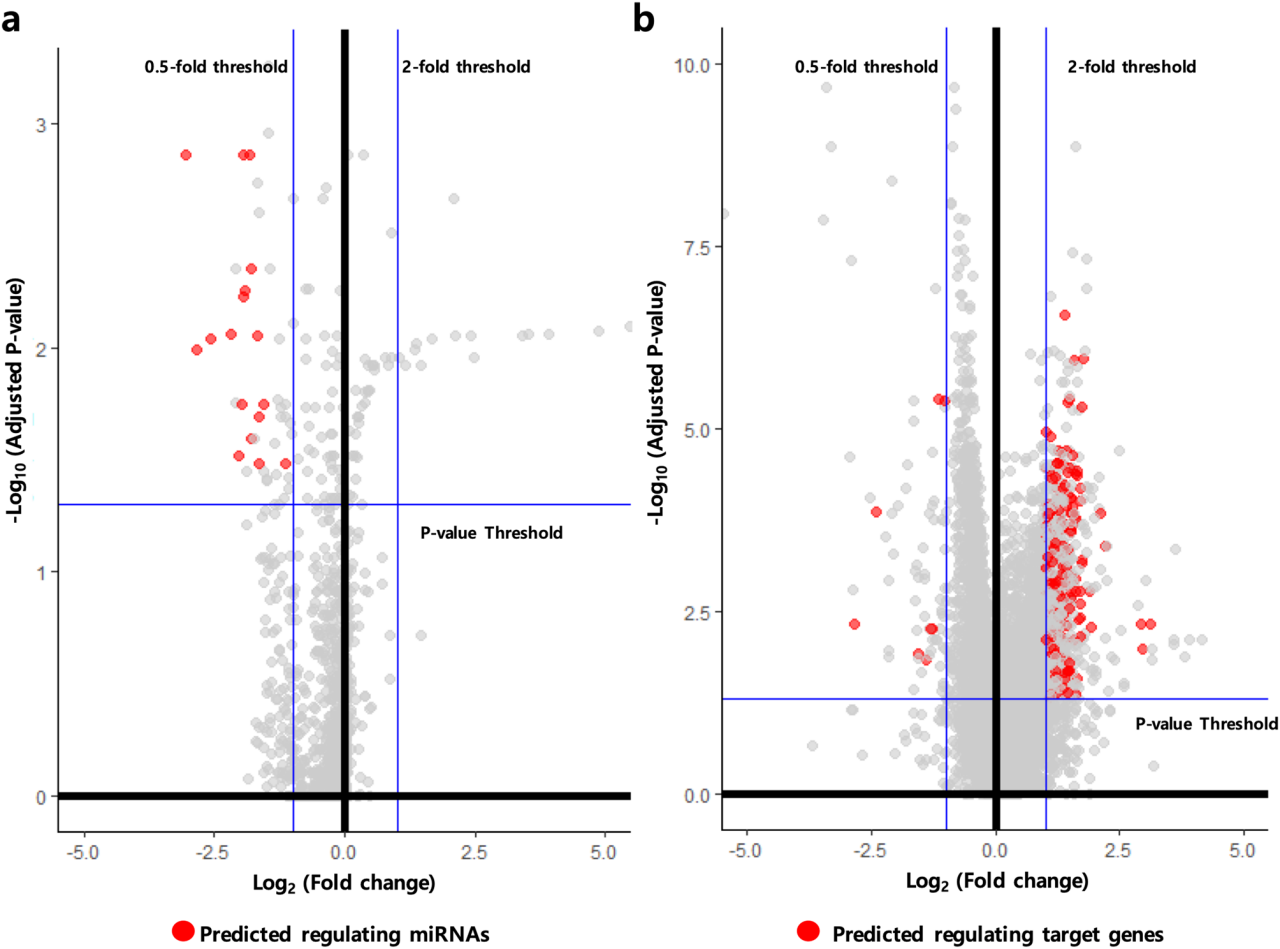
Volcano plots of (**a**) miRNAs and (**b**) genes that show the expressional fold-changes of primary tumor samples with metastasis, compared to primary tumor samples without metastasis. Each dot represents miRNA or gene with its expressional fold-change and corresponding adjusted *p* value. Red dots indicate miRNAs and genes that are identified as metastasis-initiating miRNA-target regulations. All metastasis-inhibiting miRNAs show decreased expressions, and the expressions of their target genes are mostly increased.

**Figure 3:**
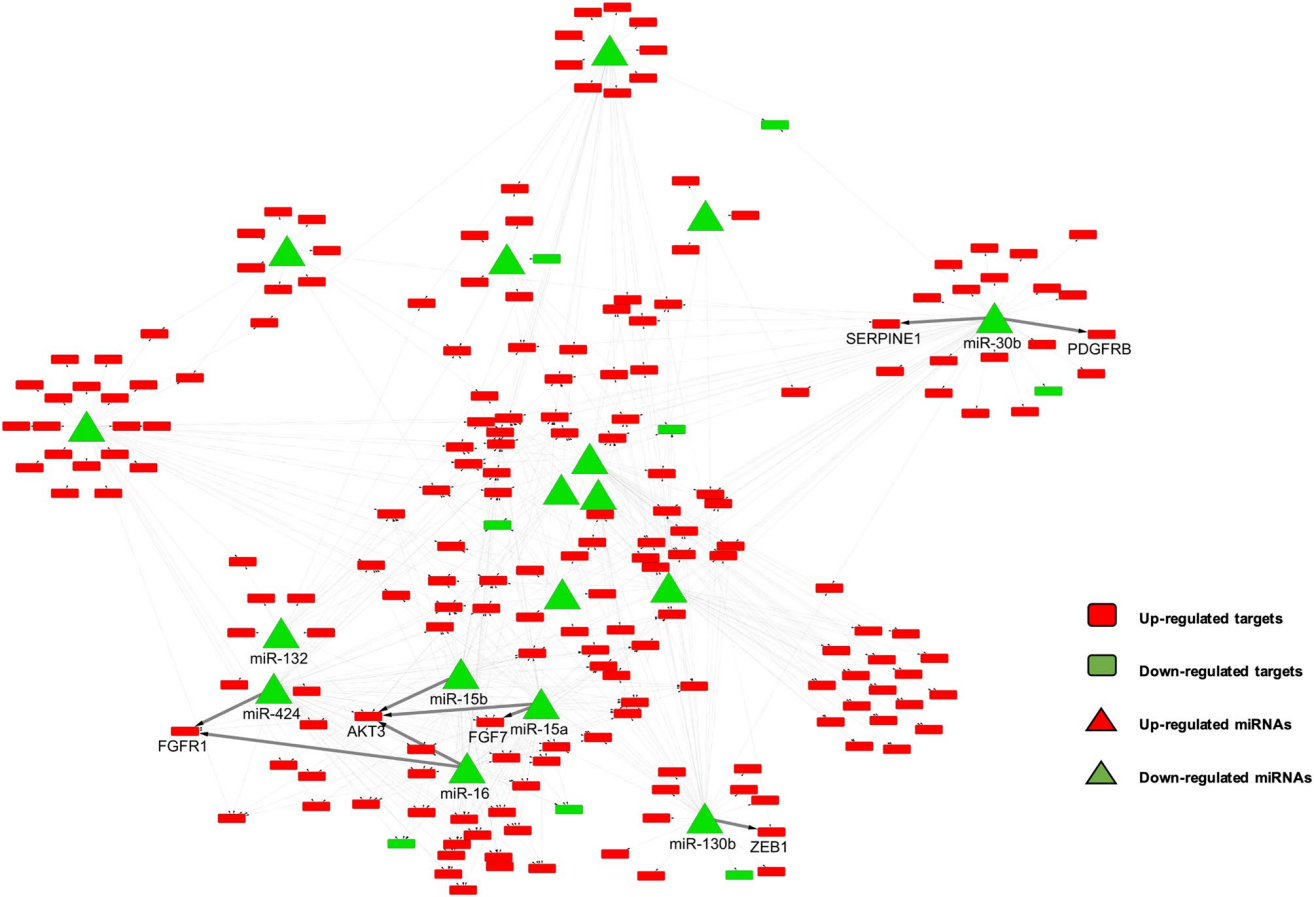
Identified metastasis-initiating miRNA-target regulations. Up-regulated DEGs indicate that they have increased expression levels in primary tumor samples with metastasis, compared to primary tumors without metastasis, while down-regulated DEmiRNAs and DEGs have decreased expressions. All miRNAs are down-regulated, and they constitute the majority of the identified miRNA-target regulations with up-regulated target genes. Selected names of previously validated miRNAs and their target genes are shown. The network diagram was illustrated using Cytoscape 3.8 (https://cytoscape.org).

**Figure 4:**
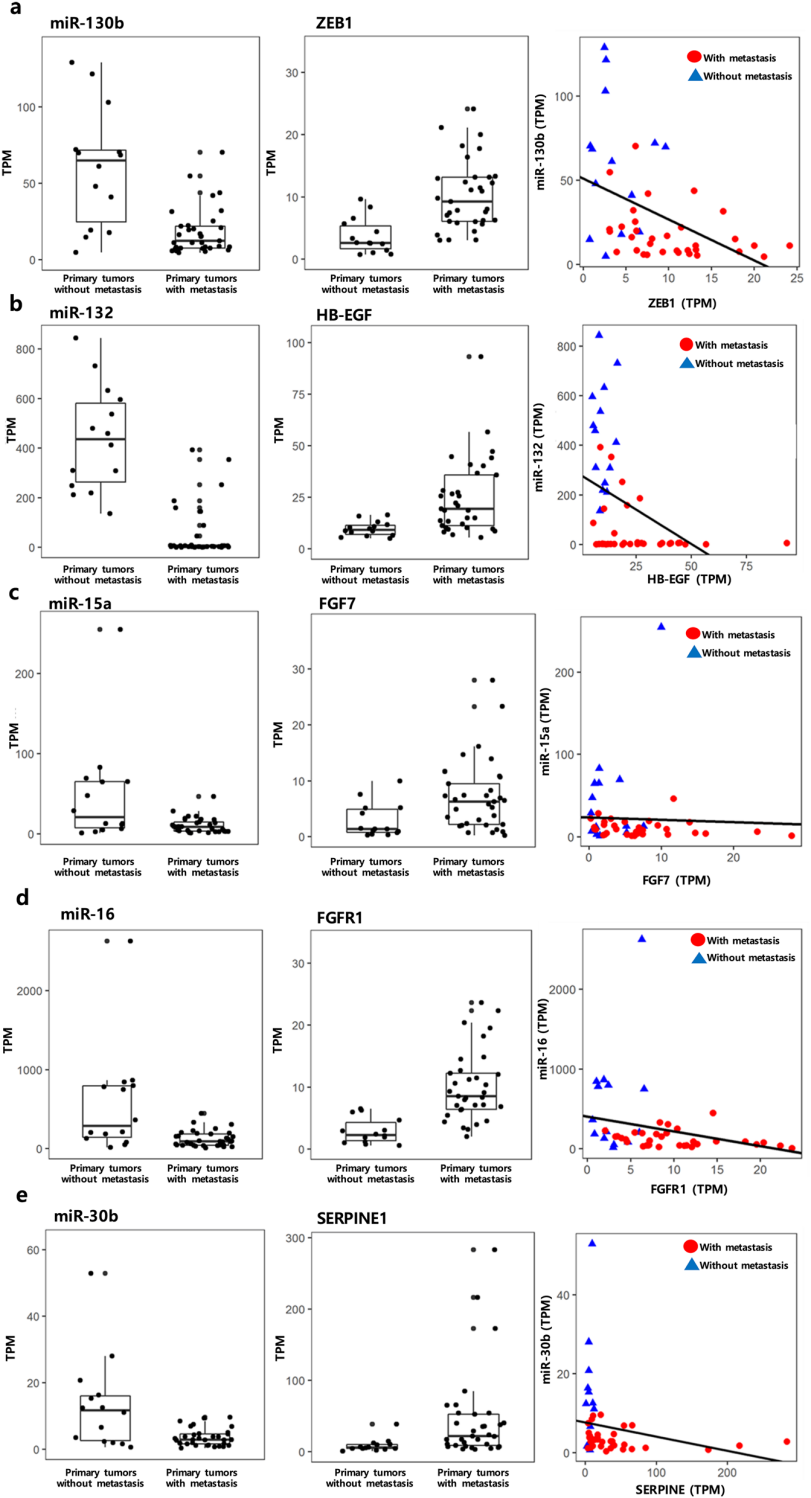
Expression levels of selected miRNAs and their target genes from the identified regulations with previously reported cases on cancer progression and metastasis. (**a**) miR-130b-ZEB1, (**b**) miR-132-HB-EGF, (**c**) miR-15a-FGF7, (**d**) miR-16-FGFR1, (**e**) miR-30b-SERPINE1.

Comparing the expression levels of miRNAs and genes of the two primary tumor sample groups, with and without metastasis, we identified 58 DEmiRNAs and 639 DEGs (Supplementary Fig. S6 online and Fig. 2). Of these, 516 metastasis-initiating miRNA-target regulations, including 17 DEmiRNAs and 198 target DEGs, were predicted (Figs. 2 and 3, see Supplementary Fig. S7 online for a high-resolution illustration of the miRNA-target network with all miRNA and gene names). We observed that all of the 17 miRNAs were down-regulated in primary tumors with metastasis. Likewise, only eight of the 198 target genes from the identified regulations were down-regulated in primary tumors with metastasis and 190 target genes were up-regulated. These results imply decreased expression of all metastasis-inhibiting miRNAs in primary tumors with metastasis, and generally increased expressions of their target genes. Previous studies reported that some of these are related with cancer progression and metastasis, where 12 of 17 miRNAs have been experimentally validated to either bind to their predicted targets or to regulate the predicted target genes in cancer or other diseases (see Supplementary Table S3 online for detailed information on the complete list of the identified 516 miRNA-target regulations). A few selected examples are illustrated in Fig. 4. We observed that miRNA-130b is down-regulated and ZEB1 is up-regulated in primary tumors with metastasis (Fig. 4a). It is known that the expression of ZEB1, a key gene associated with epithelial-mesenchymal transition, increases subsequent to deregulation of miR-130b^22^. The identified miR-132—HB-EGF regulation (Fig. 4b) has also been reported in prostate cancer cell lines^23^, where the HB-EGF, associated with cell migration and invasion, is inhibited by miR-132. We also identified the inhibition of FGF7 (a well-known fibroblast growth factor involved in tumor growth and invasion, and a potent epithelial cell-specific growth factor) by miR-15a (Fig. 4c); a previous study reported that the down-regulation of miR-15a progresses pancreatic cancer^24^. Considering the disturbed inhibition of FGF7 by miR-15a in primary tissues with metastasis, it is interesting that we also identified the miR16—FGFR regulation as a metastasis-initiating miRNA-target regulation. Our result shows down-regulation of miR16 and corresponding up-regulation of FGFR in primary tumors with metastasis (Fig. 4d), where FGFR is a receptor for FGF7 and well known to be related to proliferation and migration of endothelial cells and angiogenesis^25^. The regulation of miR-30b—SERPINE1 (Fig. 4e) is also reported to be related to gastric cancer tumorigenicity^26^, where restoring the regulation of SERPINE1 by miR-30b increases apoptosis of cancer cells in vivo.

**Figure 5:**
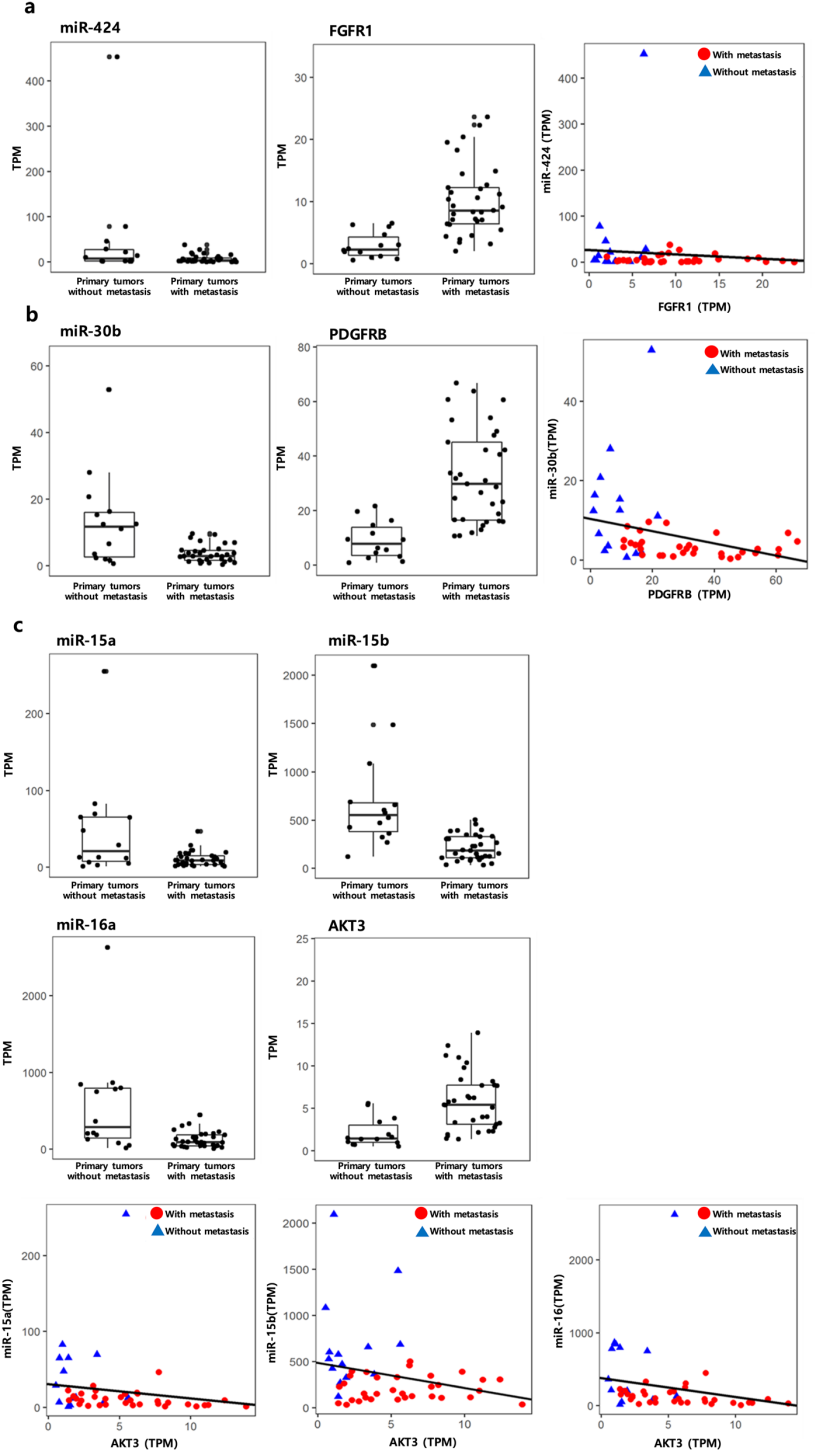
Expression levels of miRNAs and their target genes that are well-known with their relevance to colorectal cancer progression and have previously reported validations on miRNA-gene regulations. (**a**) miR-424-FGFR1, (**b**) miR-30b-PDGFRB, (**c**) regulation of AKT3 by miR-15a, miR-15b, and miR-16.

Some of the identified miRNA-target regulations from our results (Fig. 5) include genes previously identified as relevant to CRC, with experimental validations of their regulations provided in corresponding studies. FGFR1 is reported to be associated with cell proliferation, differentiation and angiogenesis in cancer^27^. Previous studies have shown that miR-424 inhibits FGFR1^28–30^, and the regulation of miR-424—FGFR1 was predicted to be metastasis-initiating from our results with down-regulated miR-424 and up-regulated FGFR1 (Fig. 5a). The PDGFRB gene plays an important role in proliferation and migration of various tumors^31^, and studies have reported that PDGFRB represses miR-30b^32^, which is also seen in this study (Fig. 5b). AKT3 is an oncogene associated with the regulation and metastasis of tumor cell survival^33^. We identified that regulation of AKT3 by miR-15a, miR-15b and miR-16 is disturbed in primary tumors with metastasis (Fig. 5c), where the miRNAs are down-regulated and AKT3 is up-regulated; this disturbed regulation of AKT by miRNAs with expressional changes has also been reported previously^34,35^.

### Identified miRNA-target regulations have a statistically significant relationship with metastasis

**Figure 6:**
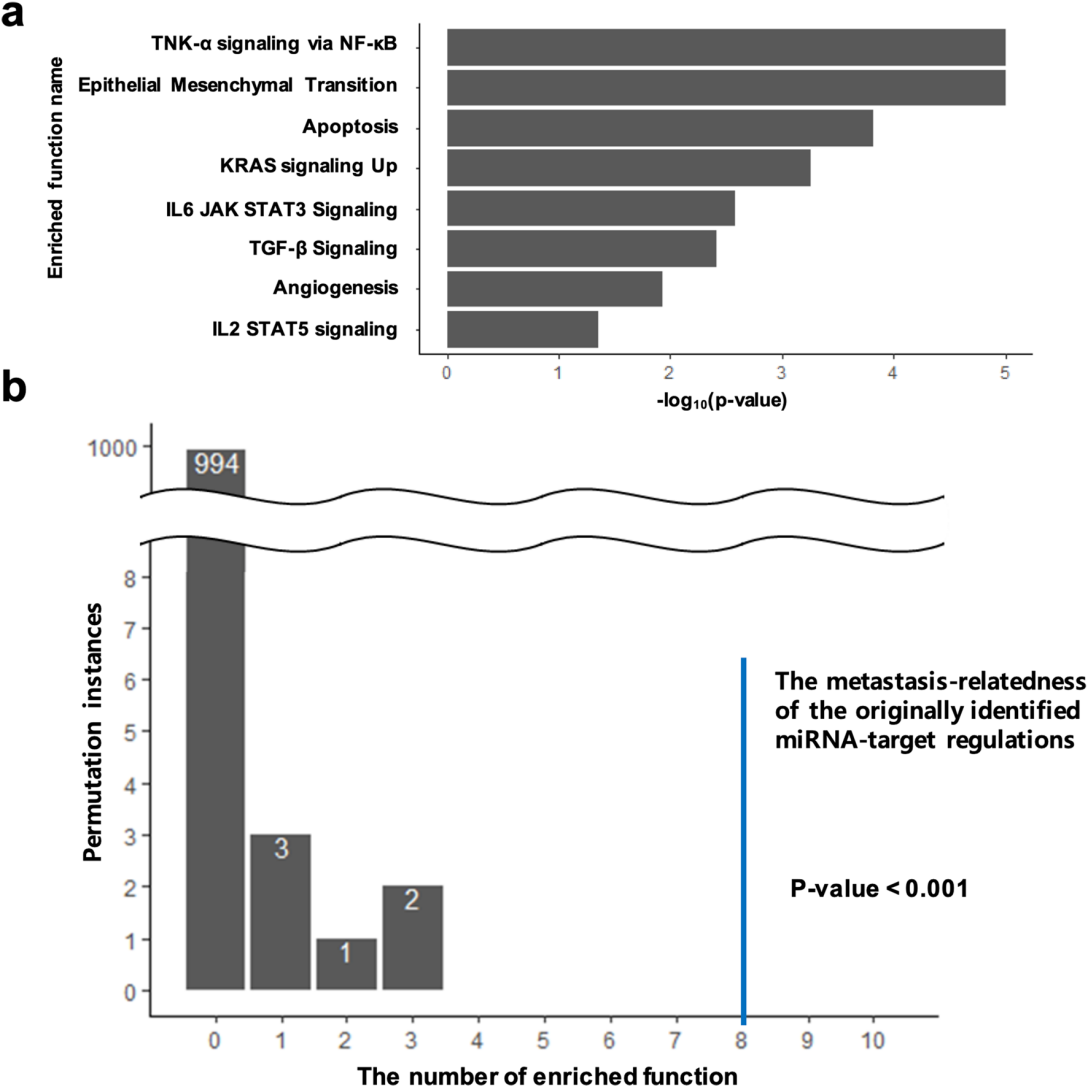
(**a**) Enriched functions of cancer progression and metastasis in the target genes of the identified miRNA-target regulations and their *p* values of statistical significance. Among the 22 metastasis and cancer-related hallmark gene sets, eight are enriched. (**b**) Evaluated statistical significance of metastasis-relatedness of the identified miRNA-target regulations by random permutation test (1,000 random permutations of sample group labels). All 1,000 instances of identifying metastasis-initiating miRNA-target regulations from randomly permuted data sets show less metastasis-relatedness than that of the identified miRNA-target regulations from the original data set (equivalent to *p* value < 0.001).

In order to evaluate the metastasis-relatedness of the identified miRNA-target regulations, we performed the functional enrichment test, as described in the Methods section. Functional enrichment analysis revealed that eight of the 22 hallmark gene sets related to cancer progression and metastasis are enriched in the target genes of the identified miRNA-target regulations (Fig. 6a), and this amount of enriched gene sets reflects the degree of metastasis-relatedness. A random permutation approach was applied 1,000 times, to evaluate its statistical significance by analyzing data with randomly shuffled sample group labels (with metastasis and without metastasis). Among the 1,000 randomly permuted instances, there was no case with metastasis-relatedness higher than or equal to the metastasis-relatedness of the original data, corresponding to *p* value < 0.001 (Fig. 6b). This statistical significance indicates that the identified miRNA-target regulations are significantly associated with the status of liver metastasis.

### Different patients’ survivals based on the patterns of the identified miRNA-target regulations

**Figure 7:**
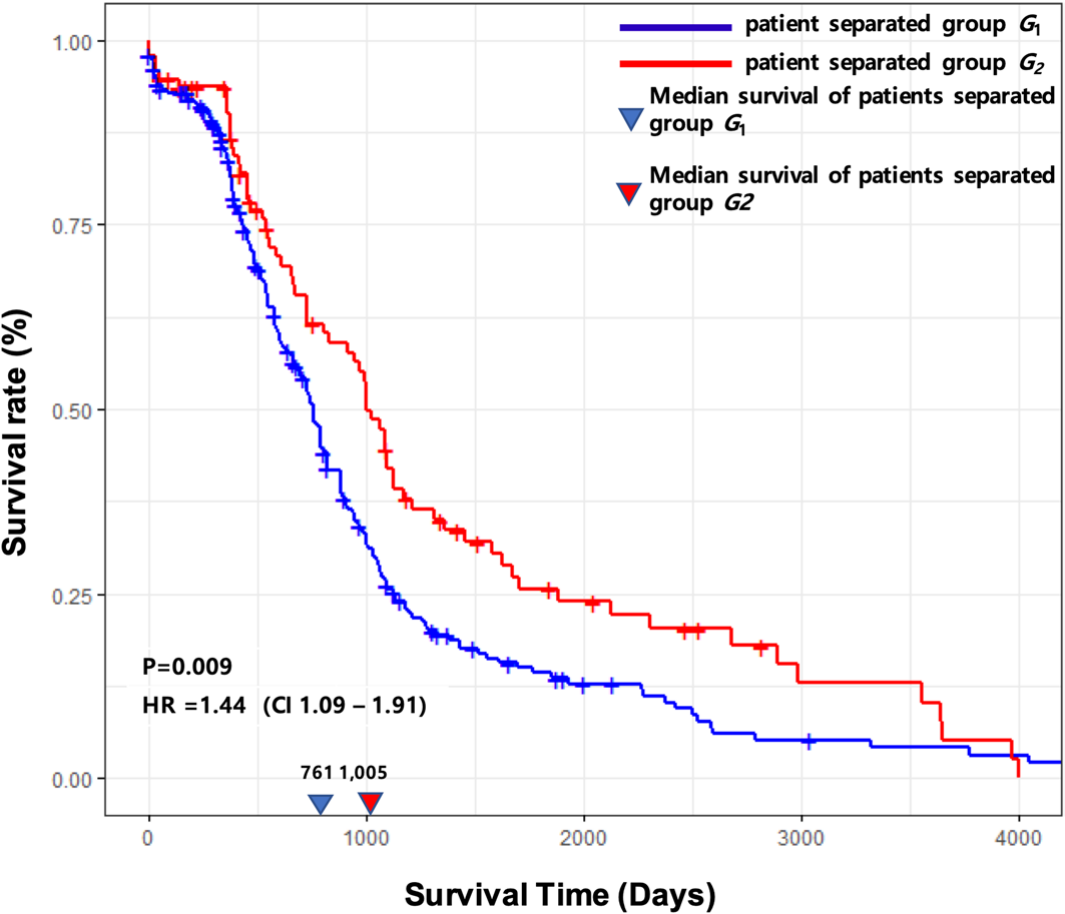
Kaplan–Meier curves of the two categorized groups of patients from the TCGA colorectal cancer, where one group (*G*_1_) has more similar miRNA-target expression profile patterns with primary tumors with metastasis than the other group (*G*_2_). The survival rates of both groups are significantly different (Hazard ratio = 1.44 (CI 1.09–1.91), *p* value = 0.009; Adjusted hazard ratio with gender, race and age = 1.35 (CI 1.06–1.90), *p* value 0.012).

From the processes described in Methods, 426 primary tumor samples of TCGA CRC data were categorized into two groups, based on the expression similarity with the identified metastasis-initiating miRNA-target regulations; one group has greater similar expression patterns to primary tumors with metastasis than the other group. Survival comparison between the two groups using the log-rank test revealed significantly differing survival rates (*p* value = 0.009, Hazard ratio = 1.44 (CI 1.09–1.91)). We also investigated whether this difference in survival is independent of other clinical characteristics by using the multivariate Cox-PH model. The adjusted hazard ratio with gender, race and age was 1.35 (CI 1.06–1.90, *p* value = 0.012), thus representing this survival difference is independent of other clinical variables. The group of patients with expression patterns more similar to the metastasis-initiating miRNA-target regulations showed median survival of 761 days, whereas the median survival of the other group was 1,005 days (Fig. 7). This significant difference of prognosis from analyzing independent data by the identified miRNA-target regulations implies that the identified regulations play important roles in the progression of CRC.

## Discussion

In the current study, we compared the expression levels of miRNAs and genes of primary tumors from CRC patients, with and without metastasis, to identify metastasis-initiating miRNA-target regulations. We identified 516 metastasis-initiating miRNA-target regulations, including 17 miRNAs and 198 target genes. Statistical analysis revealed that the identified metastasis-initiating miRNA-target regulations are strongly related to cancer progression and metastasis, with statistically significant difference. We also evaluated the clinical significance of the identified miRNA-target regulations by separating primary tumor samples from the TCGA CRC data set, based on their resemblance to the identified regulations. From the survival comparison of the two categorized patient groups, the group that exhibited more resemblance to the identified metastasis-initiating miRNA-target regulations had shorter survival periods than the group showing lesser resemblance. This analysis reveals the important clinical implications of the identified miRNA-target regulations, especially considering that liver metastasis is a major factor that affects the survival of CRC patients. The identified metastasis-initiating miRNA-target regulations include molecular mechanisms that were reported to be associated with cancer progression and metastasis in different cancer types by previous studies, as well as regulations with well-known genes for CRC. The patterns in expression changes of miRNAs and their regulated genes in our data analysis are also consistent with that of previous findings. Some other novel regulations identified from the current study are potential candidates for future experimental validations. While our results present promising metastasis-initiating miRNA-target regulations, it has limitation of using only expression data for miRNAs and genes. Other genetic features that can contribute to the metastasis of colorectal cancer include DNA somatic mutations and copy number variations (CNVs). Hence, by evaluating somatic mutations and CNVs along with expressions for analysis, further coverages of regulations between miRNA and target genes can be considered in progressing tumors and initiating metastasis. Another limitation is that we used predicted miRNA-target relationship information in identifying target genes of miRNAs, including many miRNA-target relationships that have not yet been experimentally verified. Nevertheless, we believe that the identified miRNA-target regulations from this study can provide meaningful clues and guidance for researching and experimentally validating metastasis-initiating miRNA-target regulations for CRC.

## Supplementary information

Supplementary file1

Supplementary file2

Supplementary file3

## Data Availability

The datasets generated and analyzed during the current study are available from the corresponding author on reasonable request.
