# Supplementary material for: Identifying metastasis-initiating miRNA-target regulations of colorectal cancer from expressional changes in primary tumors
Source: Sci Rep. 2020 Sep 10;10:14919. doi: 10.1038/s41598-020-71868-0 (PMC7484763; doi:10.1038/s41598-020-71868-0)
Supplement: Supplementary file 1 — Supplementary file1 [file 41598_2020_71868_MOESM1_ESM.docx]

**Supplementary Information**

**Identifying metastasis-initiating miRNA-target regulations of colorectal cancer from expressional changes in primary tumors**

**Jongmin Lee**^1*^**, Hye Kyung Hong**^2*^**, Sheng-Bin Peng**^3*^**, Tae Won Kim**^2^**, Hee Cheol Kim**^2^**, Seong Hyun Yun**^2^**, Jiangang Liu**^3^**, Philip J. Ebert**^3^**, Amit Aggarwal**^3^**, Woo Yong Lee**^2^**, Sungwon Jung**^1,4C^**, Yong Beom Cho**^2C^

^1^Gachon Institute of Genome Medicine and Science, Gachon University Gil Medical Center, Incheon, Republic of Korea

^2^Department of Surgery, Samsung Medical Center, Sungkyunkwan University School of Medicine, Seoul, Republic of Korea

^3^Eli Lilly and Company, Indianapolis, USA

^4^Department of Genome Medicine and Science, Gachon University College of Medicine, Incheon, Republic of Korea

*: Equally contributed to this work

c: To whom correspondence should be addressed

**Supplementary Table S1.** Demographic and clinical landscape of the 47 CRC patients. The two groups of patients with and without metastasis did not show statistically significant differences in ages, sex, tumor locations, and cell differentiations, and the corresponding p-values from the chi-square test are given in parentheses. Cell differentiations were categorized for adenocarcinoma and mucinous carcinoma for the statistical test. Tumor stages, invasion status, and MSI status were not considered for statistical evaluation as they are directly or closely related to the status of metastasis.

|  |  | Non-meta Primary | With meta primary |
| --- | --- | --- | --- |
| Number |  | 14 | 33 |
| Age  (p-value = 0.606153) | <=60 >60 | 10 (71.4%) 4 (28.6%) | 21 (63.6%) 12 (36.4%) |
| Sex  (p-value = 0.924292) | Male Female | 7 (50.0%) 7 (50.0%) | 16 (48.5%) 17 (51.5%) |
| Tumor Location  (p-value = 0.123051) | Colon Rectum | 13 (92.9%) 1 (7.1%) | 24 (72.7%) 9 (27.3%) |
| Cell Differentiation  (p-value = 0.522957) | Adenocarcinoma W/D M/D P/D Mucinous carcinoma | 1 (7.1%) 11 (78.7%) 1 (7.1%) 1 (7.1%) | 3 (9.1%) 27 (81.8%) 2 (6.1%) 1 (3.0%) |
| Stage | II III IV | 7 (50.0%) 7 (50.0%) 0 (0%) | 0 (0%) 0 (0%) 33 (100%) |
| Invasion | Lymphatic Perineural Vascular | 5 (35.7%) 3 (21.4%) 3 (21.4%) | 18 (54.5%) 20 (60.6%) 16 (48.5%) |
| MSI Status | MSS MSI Undesecribed | 11 (78.6%) 3 (21.4%) 0 (0%) | 32 (97.0%) 0 (0%) 1 (3.0%) |

W/D, well differentiated; M/D, moderately differentiated; P/D, poorly differentiated; MSI, microsatellite instability; MSS, microsatellite stable

**Supplementary Table S2.** The 22 selected hallmark gene sets related to cancer progression and metastasis

| **Gene set name** | **Number of genes** | **Brief description** |
| --- | --- | --- |
| TNF-α signaling via NF-κb | 200 | Genes regulated by NF-kB in response to TNF |
| WNT beta catenin signaling | 42 | Genes up-regulated by activation of WNT signaling through accumulation of beta catenin CTNNB1 |
| TGF-β signaling | 54 | Genes up-regulated in response to TGFβ1 |
| IL6 JAK STAT3 signaling | 87 | Genes up-regulated by IL6 via STAT3 |
| DNA repair | 150 | Genes involved in DNA repair |
| Apoptosis | 161 | Genes mediating programmed cell death (apoptosis) by activation of caspases |
| Notch signaling | 32 | Genes up-regulated by activation of Notch signaling |
| Interferon-α response | 97 | Genes up-regulated in response to alpha interferon proteins |
| Interferon-γ response | 200 | Genes up-regulated in response to IFNγ |
| Hedgehog signaling | 36 | Genes up-regulated by activation of hedgehog signaling |
| PI3K AKT MTOR signaling | 105 | Genes up-regulated by activation of the PI3K/AKT/mTOR pathway |
| MTORC1 signaling | 200 | Genes up-regulated through activation of mTORC1 complex |
| E2F targets | 200 | Genes encoding cell cycle related targets of E2F transcription factors |
| MYC targets v1 | 200 | A subgroup of genes regulated by MYC - version 1 |
| MYC targets v2 | 58 | A subgroup of genes regulated by MYC - version 2 |
| Epithelial mesenchymal transition | 200 | Genes defining epithelial-mesenchymal transition, as in wound healing, fibrosis and metastasis |
| Glycolysis | 200 | Genes encoding proteins involved in glycolysis and gluconeogenesis |
| P53 pathway | 200 | Genes involved in p53 pathways and networks |
| Angiogenesis | 36 | Genes up-regulated during formation of blood vessels (angiogenesis) |
| IL2 STAT5 signaling | 200 | Genes up-regulated by STAT5 in response to IL2 stimulation |
| KRAS signaling up | 200 | Genes up-regulated by KRAS activation |
| KRAS signaling down | 200 | Genes down-regulated by KRAS activation |

**
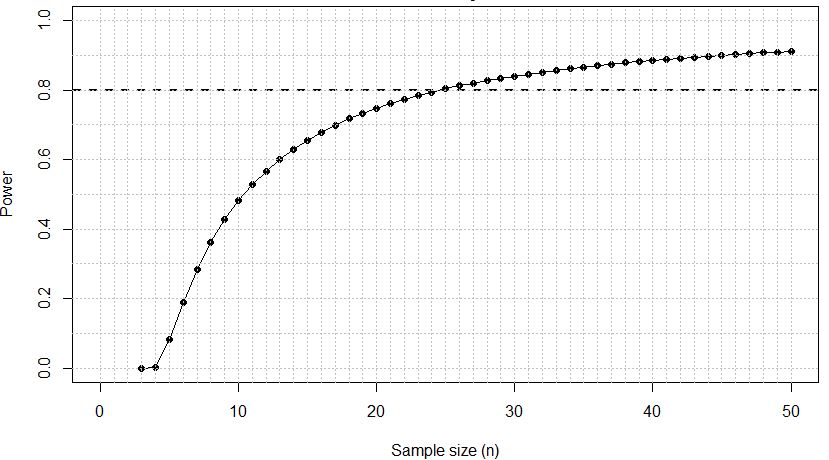
**

**Supplementary Figure S1.** The result of power analysis for whole-transcriptome data using the ssizeRNA (Bi and Liu, BMC Bioinformatics 17, 146 (2016)) R package. Power analysis was conducted based on average expressions and variations from our data, and parameters of two-fold changes and FDR = 0.05 was used for simulations. Other parameters were used as defaults. The suggested power value of 0.8 by the developers can be achieved with about 24 samples, while the two sample groups of our data have 14 and 33 samples, implying that the data configuration of our data set is on borderline of meeting the statistical suggestion.


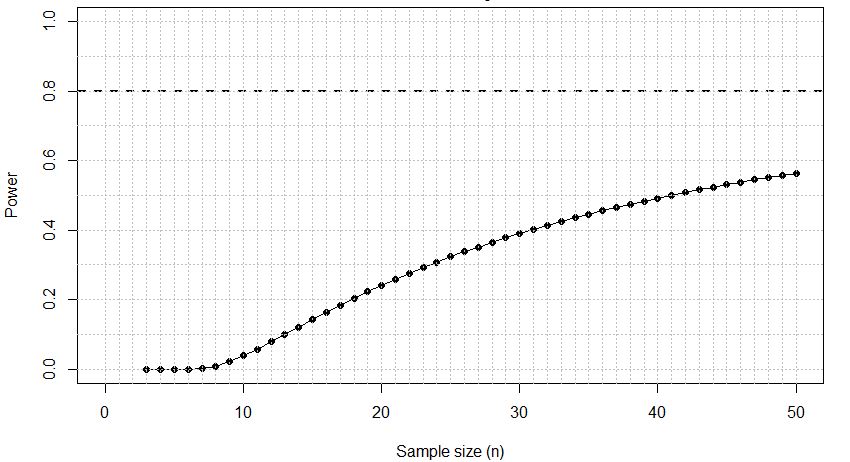


**Supplementary Figure S2.** The result of power analysis for miRNA expression data using the ssizeRNA R package. Power analysis was conducted based on average expressions and variations from our data, and parameters of two-fold changes and FDR = 0.05 was used for simulations. Other parameters were used as defaults. The suggested power value of 0.8 by the developers cannot be achieved with less than 50 samples, while this low statistical power of miRNA data can be due to much lower amounts of sequence reads from miRNAs than mRNAs in general as shown in Supplementary Figure S3.


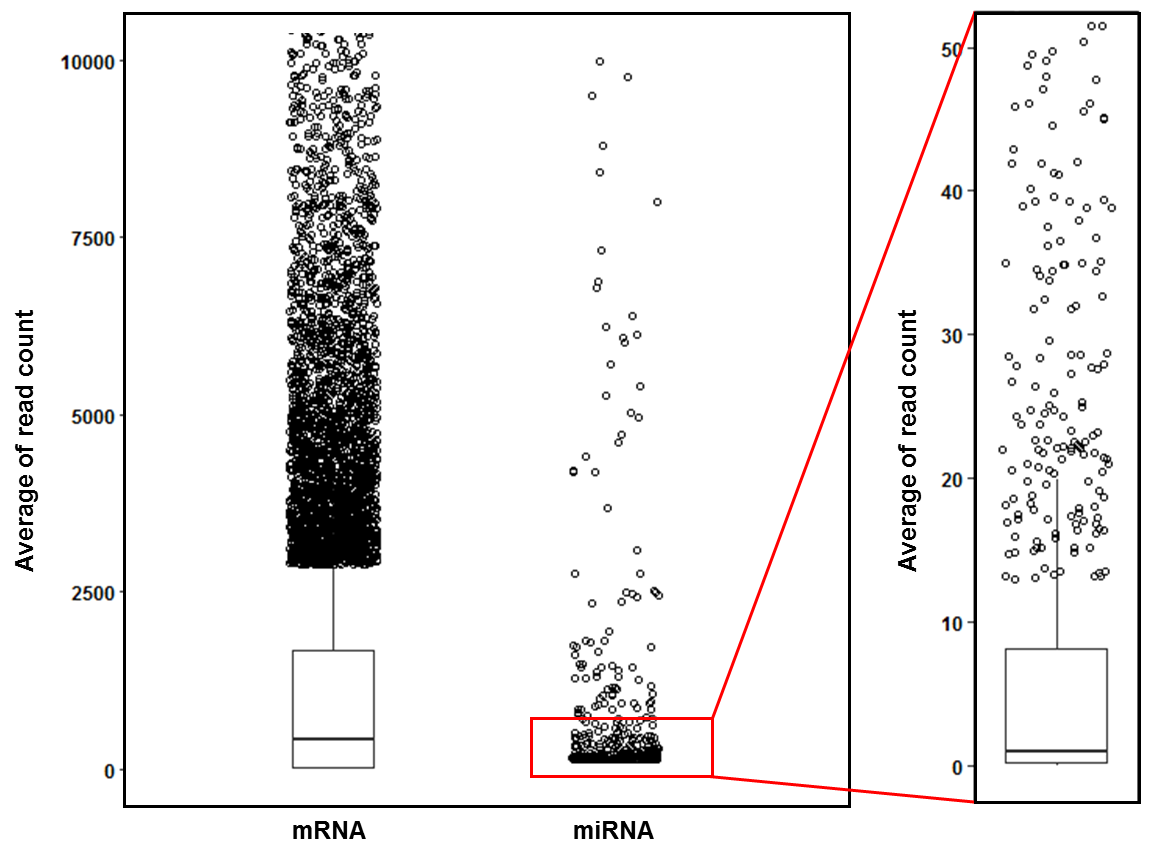


**Supplementary Figure S3.** The average read counts for 23,956 genes (mRNA) and 2,669 miRNAs. Even with considering the lower targeted sequencing depth of miRNA (10 million reads per sample) compared to that of RNA-seq (50 million reads per sample), miRNAs generally show much lower expressions with lower variations than genes. Deeper targeted sequencing depth or more samples can be necessary for miRNAs to achieve comparable statistical power with RNA-seq data, where the availability of more sample materials should be considered.

**Supplementary Figure S4.** The schematic illustration of evaluating the statistical significance of the metastasis-specificity for the identified miRNA-target regulations by random permutation approach. From the original analysis, the identified miRNA-target regulations showed enrichment in eight of the 22 metastasis-related functions. In order to estimate its statistical significance, 1,000 randomly permuted analyses were conducted to constitute a random permutation test. For each randomly permuted analysis, the metastasis status information on samples was randomly re-assigned and the entire process of identifying differential expressions, miRNA-target regulations, and functional enrichment of target genes were performed. Each analysis with a randomly shuffled data gives the number of enriched metastasis-related functions at the end, and 1,000 observations on the number of enriched metastasis-related functions were collected from 1,000 permuted analyses. This gives the empirical null distribution on the number of enriched metastasis-related functions by random chance, where the frequency of observations that showed eight or more enriched metastasis-related functions among the 1,000 permuted analyses can be considered as a statistical significance p-value for the result from the original analysis. As none of the 1,000 permuted analyses showed eight or more enriched metastasis-related functions from our experiment, it represents that observing enrichment on eight out of the 22 metastasis-related functions from the original analysis achieves statistical significance of a p-value < 0.001 by the random permutation test.

**Supplementary Figure S5.** Three cases of determining the representative ranges of miRNA/gene expressions from our data set. For each miRNA or gene, its representative expression ranges within a sample group (*woM* and *wM*) are defined with their mean expression ± standard deviation values from the z-transformed expressions of corresponding sample groups, but differently according to their overlaps. **(a)** When there is no overlap between the two expression ranges *R_woM_* and *R_wM_*, each expression range is defined as mean expression ± standard deviation. **(b)** and **(c)** When there is overlap between *R_woM_* and *R_wM_*, their ranges are separated at the center of the overlap so that an expression value can belong within only one range.

**Supplementary Figure S6.** Expression heatmaps of the identified DEmiRNAs and DEGs. DEmiRNAs and DEGs with many zero expression values are not displayed in these figures.

[Supplementary Figure S7 is separately available online.]

**Supplementary Figure S7.** The high-resolution network illustration of the identified metastasis-initiating miRNA-target regulations. The names of miRNAs and their target genes are presented with individual nodes. The network diagram was illustrated using Cytoscape 3.8 (https://cytoscape.org).
