# Supplementary figures and images for: Identifying metastasis-initiating miRNA-target regulations of colorectal cancer from expressional changes in primary tumors
Source: Sci Rep. 2020 Sep 10;10:14919. doi: 10.1038/s41598-020-71868-0 (PMC7484763; doi:10.1038/s41598-020-71868-0)

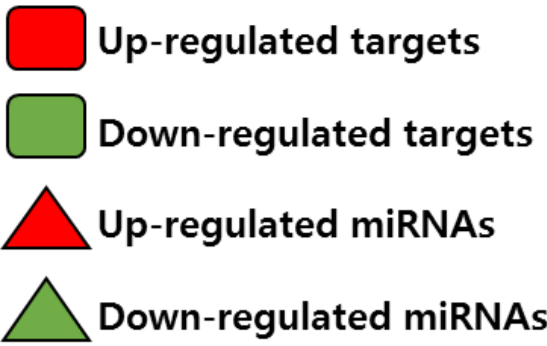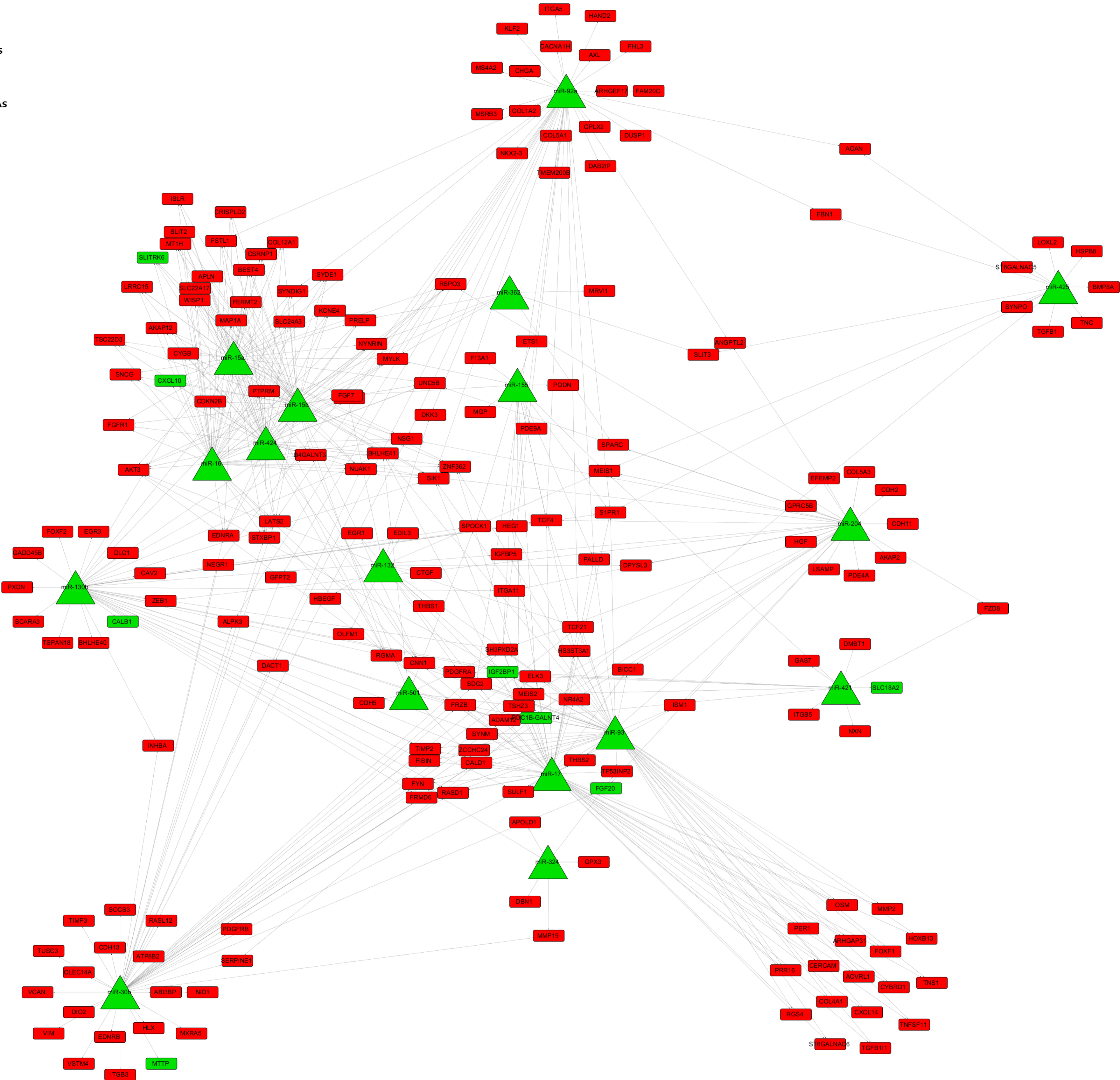

Supplement: Supplementary file 2 — Supplementary file2 [file 41598_2020_71868_MOESM2_ESM.pdf]
